# Pediatric Cervical Lymphadenitis: Etiology, Clinical Presentation, and Antimicrobial Resistance

**DOI:** 10.1155/ijpe/5154191

**Published:** 2025-05-09

**Authors:** Mahmoud Khodabandeh, Zahra Jam, Aryan Banai Shahani, Mahsa Soti Khiabani

**Affiliations:** ^1^Department of Infectious Diseases, Pediatrics Center of Excellence, Children's Medical Center, Tehran University of Medical Sciences, Tehran, Iran; ^2^Children's Medical Center, Pediatrics Center of Excellence, Tehran, Iran; ^3^Department of Pediatrics, Children's Medical Center, School of Medicine, Tehran University of Medical Sciences, Tehran, Iran; ^4^School of Medicine, Tehran University of Medical Sciences, Tehran, Iran

**Keywords:** antibiotic resistance, cervical, children, lymphadenitis, treatment

## Abstract

**Background:** Cervical lymphadenitis is prevalent in children. Several viruses and bacteria can cause cervical lymphadenitis. *Staphylococcus aureus* and *Streptococcus pyogenes* are known to predominate as bacterial causes. Choosing the effective antibiotic regimen to treat cervical lymphadenitis is difficult because of temporal and geographical variations in its etiologies and antibiotic resistance. We aim to elucidate the etiologies, treatment, and outcomes of cervical lymphadenitis in children in Iran.

**Methods:** A total of 113 patients admitted to the Children's Medical Center in Iran were included in this retrospective cross-sectional study. Patients under 18 years in medical records were evaluated for demographics, signs and symptoms, lymph node aspiration or surgical drainage culture and antibiogram results, type and duration of treatment, complications, treatment failure, and patient discharge instructions.

**Results:** Patients' mean age was 3.5 years (SD: 2.9; range: 3 months to 13 years), and 70 (62%) were male. Of 113 patients, 38 (34%) had a prior history of upper respiratory tract infection (URI), 2 (1.7%) had dental caries, 1 (0.9%) had Hodgkin's lymphoma, and 72 (64.1%) patients did not have any accompanying illnesses in presentation. The most common clinical manifestation was neck swelling or erythema (99.1%), followed by fever (73%), neck pain (30%), and torticollis (9%). Twenty-one (18.5%) patients underwent cervical lymph node aspiration, and 7 (6%) underwent surgical incision and drainage, of which 17 (61%) had a positive culture. *Staphylococcus aureus* was isolated in 16 (94%) cases. No positive culture was reported regarding fungi and acid-fast bacilli. Regarding their antibiogram reports, the lowest resistance rates were to vancomycin, cotrimoxazole, and oxacillin (6% each), followed by clindamycin and erythromycin (12% each) and penicillin (94%). The mean duration of hospitalization was 6 days (SD: 3.2; range: 2–22 days). Thirty-three (29%) patients underwent surgical drainage along with antibiotic therapy.

**Conclusion:** Cervical lymphadenitis was prevalently accompanied by URI. Swelling and erythema in the neck were the most common clinical manifestations. The most common isolated organism was *Staphylococcus aureus*. We did not find Streptococci, which might be due to the beta-lactam usage before hospital admission. Most of the patients were treated with clindamycin during hospitalization. However, resistance to clindamycin was higher than that of other antibiotics effective against Staphylococci and Streptococci, like oxacillin. We recommend considering this resistance pattern in choosing antibiotics to prevent treatment failure and reduce the need for surgery.

## 1. Introduction

Cervical lymphadenopathy is a very common presentation in children [1]. Enlargement of lymph nodes can have infectious, neoplastic, rheumatologic, or pharmacologic causes. However, lymphadenitis is considered to result from an inflammatory process. At the time of presentation, lymphadenitis can have various symptoms such as lymph node enlargement, fever, malaise, erythema, edema, and pain in the overlying skin [2, 3]. Fever and torticollis are considered the most common presentations of cervical lymphadenitis [4].

Among its etiologies, reactive hyperplasia resulting from bacterial or viral infections is the most common cause of cervical lymphadenitis [5, 6]. Several upper respiratory tract viruses, along with Epstein–Barr virus (EBV), Cytomegalovirus (CMV), and human immunodeficiency virus (HIV) can cause cervical lymphadenitis [5]. Additionally, among the bacterial causes, *Staphylococcus aureus* and *Streptococcus pyogenes* are responsible for the majority of the cases. Furthermore, they are most common in children under 4 years [6, 7]. Following these bacteria, *Bartonella* and mycobacteria, predominantly, nontuberculous mycobacteria can also lead to lymphadenitis [7, 8]. Moreover, anaerobic bacteria are prevalently detected in the aspiration culture and are mostly seen in adolescents or people with periodontal diseases [6, 9].

Usually, cervical lymphadenitis will resolve on its own throughout 4–6 weeks, with no special treatment. In the remaining cases, cervical lymphadenitis may progress and undergo liquefactive necrosis and result in acute suppurative cervical lymphadenopathy or retropharyngeal and parapharyngeal space abscesses, which may need more invasive interventions [10].

A lymph node needle aspiration can be an effective strategy in cases not responding to antibiotics after 48–72 h or in patients who have localized fluctuations or swelling in their physical exam, especially in small, unilocular abscesses, serving as an initial intervention to prevent infection progression and further invasive procedures [11–15], or it can help with determining the etiology of lymphadenitis by enabling microbiological and cytological analysis [14, 16].

However, surgical incision and drainage are indicated if the patient's symptoms fail to improve or worsen or the abscess recurs despite needle aspiration and antibiotic therapy [7, 11]. Additionally, surgical drainage might be considered in larger, multilocular, or deeply located abscesses [15] along with nontuberculous mycobacterial lymphadenitis [17]. Nevertheless, the majority of cases resolve following antibiotic therapy alone, and some studies have not found that surgical interventions are superior to antibiotic therapy [4, 14, 18].

Choosing the right antibiotic regimen to treat cervical lymphadenitis is vital because of the everchanging patterns of its infectious causes and antibiotic resistance. More specifically, some studies suggest an increase in the prevalence of methicillin-resistant *Staphylococcus aureus* (MRSA) as the cause of infections and neck abscesses in children [19, 20]. However, in recent studies, no such trend has been observed [21, 22]. Therefore, there seems to be temporal and geographical variation in etiologies and the treatment of choice in pediatric cervical lymphadenitis.

Considering the above statements, concerning Iran, treatment options and outcomes of cervical lymphadenitis remain unclear. Therefore, the purpose of this study is to evaluate the etiologies, treatment, and outcomes of cervical lymphadenitis in children in Iran.

## 2. Methods

In this retrospective cross-sectional study, a total of 113 patients, hospitalized with a diagnosis of cervical lymphadenitis, in the Children's Medical Center in Iran, from 2018 to 2022 were included. Patients under 18 years, with a diagnosis of infectious cervical lymphadenitis, were included, and the diagnosis was based on the expert opinion of the center's pediatric infectious diseases subspecialist. Patients were excluded if their diagnosis was changed throughout hospitalization and paraclinical evaluation or if they left the hospital before adequate diagnostic and therapeutic measures. The data were collected from the hospital data archive.

Patient demographics, including age, sex, and prior use of antibiotics, were gathered. Presenting signs and symptoms, their onset and duration, and accompanying diseases were also recorded. In cases in which lymph node aspiration culture or surgical incision and drainage was conducted, the result of the culture, the isolated organism, the antibiogram report, and also the blood culture results were evaluated. Finally, the type and duration of treatment, complications, treatment failure, and patient discharge instructions were also recorded by reviewing physician orders written in medical records. The gathered information was entered in preprepared questionnaires.

The information entered in the questionnaires was analyzed using SPSS (Statistical Package for the Social Sciences) version 22 software (SPSS Inc). Quantitative and qualitative data were described by mean (standard deviation, range) and frequency (percentage). Categorical variables were compared by the chi-squared test. Quantitative variables were compared by independent sample *T*-test. A *p* value < 0.05 was considered significant in this study. Tehran University of Medical Sciences has approved this study (ethics code: IR.TUMS.CHMC.REC.1397.049).

## 3. Results

The studied population comprised 43 (38%) girls and 70 (62%) boys. Patient characteristics are shown in Table 1. The mean age of the patients was 3.5 years (SD: 2.9; range: 3 months to 13 years). Regarding their age, there were a total of 35 (31%) children under 1 year old, 33 (29%) between 1 and 3, 26 (23%) between 3 and 6, and 19 (16%) above 6 years old. Overall, 22 (19%) of the hospitalizations were in spring, 28 (25%) in summer, 29 (26%) in fall, and 34 (30%) in winter.

Overall, 38 (34%) patients had a prior history of upper respiratory tract infection (URI). Two (1.7%) had dental caries documented in their records. One (0.9%) had Hodgkin's lymphoma. Seventy-two (64.1%) patients did not have any accompanying illnesses in presentation (Table 1).

Imaging was performed on patients who were suspected of having abscesses. The primary modality used was ultrasound, which helped assess the size, localization, and presence of a fluid collection within the lymph node. In some cases, a CT scan or MRI was used for deeper or more complicated abscesses.

### 3.1. Clinical Manifestations

Neck pain, fever, swelling and erythema, and torticollis constituted the reported manifestations at the time of hospital admission. Neck swelling or erythema (99.1%) was most commonly observed. Fever (73%) was followed by neck pain (30%) and torticollis (9%). The mean duration of symptoms before hospital admission was 5.8 days (SD: 5; range: 1–30).

### 3.2. Etiologies

Of 113 cases, 21 (18.5%) underwent cervical lymph node aspiration. Seven (6%) more cases underwent surgical incision and drainage because of needle aspiration failure. Of the 28 cases, 17 (61%) had a positive culture. Among these, 16 (94%) cases had *Staphylococcus aureus* as the isolated organism. *Staphylococcus epidermidis* growth was observed in only one of the cultures. In 17 (15%) of 113 cases, culture was done regarding fungi and acid-fast bacilli, and no positive culture was reported. In 111 (98%) cases, blood culture was conducted, of which no positive result was reported (Table 2).

Twenty-one (18.5%) patients underwent cervical lymph node aspiration, and 7 (6%) underwent surgical incision and drainage, of which 17 (61%) had a positive culture. Of 17 positive aspiration or drainage culture results, an antibiogram was requested to determine sensitivity and resistance to 6 antibiotics, including clindamycin, vancomycin, erythromycin, penicillin, oxacillin, and cotrimoxazole. Penicillin had the highest resistance rate, 94%, followed by clindamycin and erythromycin (12% each). The lowest resistance was to vancomycin, cotrimoxazole, and oxacillin, which was 6% in all three instances (Table 3).

### 3.3. Therapeutic Measures

The mean duration of hospitalization was 6 days (SD: 3.2; range: 2-22 days). In 85 (71%) cases, only antibiotic therapy was administered. In addition to antibiotic therapy, 21 (18.5%) patients underwent cervical lymph node aspiration. Seven (6%) more cases underwent surgical incision because of needle aspiration failure.

**Figure 1:**
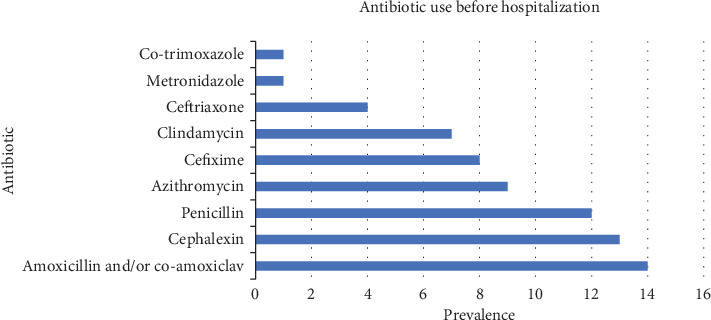
Antibiotic use before hospitalization.

Out of 113 hospitalized cases, 69 (61%) people had used antibiotics before hospitalization. The most commonly used antibiotic was amoxicillin and/or coamoxiclav, which was used in 14 (12%) patients. The antibiotics ceftriaxone and metronidazole were the least prescribed ones (Figure 1). The mean duration of antibiotic use before hospitalization was 4.6 days (SD: 3.3; range: 0–14).

**Figure 2:**
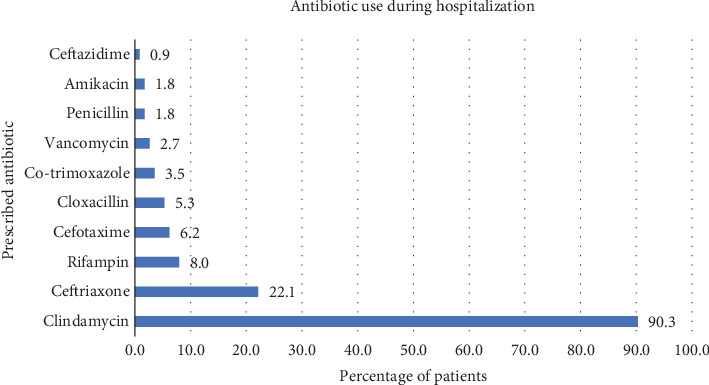
Antibiotic use during hospitalization.

Throughout hospitalization, 3 patients had their antibiotic regimen changed according to culture results. In 2 (1.7%) patients, the initial antibiotic was clindamycin, and in the other one (0.9%), it was oxacillin. Clindamycin was most widely used during hospitalization and was prescribed in 102 (90%) cases. Ceftazidime was the least-used antibiotic with a prevalence of 1 (0.9%) (Figure 2).

### 3.4. Outcomes

In patients undergoing needle aspiration or surgical drainage, the mean hospital stay was 8.8 days, the duration of symptoms was 8.3 days, and the duration of antibiotic therapy before hospitalization was 6 days. These outcomes were significantly higher than patients only receiving medical therapy, with a mean hospital stay of 5.3 days (*p* value < 0.001), symptoms lasting a mean length of 4.9 days (*p* value = 0.001), and using antibiotics 3.8 days before hospitalization (*p* value = 0.022).

## 4. Discussion

In our study, we found a male predominance in children with cervical lymphadenitis. Some studies support our data [4, 19, 21, 23, 24], but other studies have found a female predominance [10, 18, 25], and one has found no significant gender difference [14]. Additionally, we found a higher prevalence of cervical lymphadenitis in children less than 6 years old, infants being the largest group among them. Although suggesting the same overall trend, children older than 1 year comprise the largest group in previous studies [4, 19, 21, 23].

We found that hospitalizations were more common during winter, followed by fall, which another study supports [4]. This finding could be because of a higher incidence of URI in colder seasons of the year. It could be that URI can predispose patients to cervical soft tissue infections [3]. Therefore, we suggest physicians to more often include cervical lymphadenitis in the differential diagnosis in colder seasons of the year.

We also found that the most common clinical manifestations were neck swelling or erythema, followed by fever and neck pain, and torticollis was the least common manifestation. Despite some variations in clinical manifestations, other studies support our findings by coming across neck swelling and fever as the most common manifestations and torticollis absent in more than half of the cases [4, 9, 10].

According to this study, the most common accompanying illness was URI. Therefore, in patients with a history of URI presenting with neck swelling, erythema, or pain, suspecting cervical lymphadenitis earlier and starting antibiotic treatment can lead to lower rates of complications, less need for surgery, and shorter hospital stays.

Moreover, 61% of lymph node needle aspiration or surgical drainage cultures were positive, consistent with previous findings of 36%–87% [4, 10, 14, 18, 26–29]. Also, all of the cultures for acid-fast bacilli and fungi were negative. Our results are consistent with the study led by Neff et al., in which they found that despite being widely ordered by surgeons, the majority of acid-fast bacilli, fungal, or anaerobic cultures do not yield a positive result [9].

We found that the most common isolated organism was *S. aureus* (positive in 57% of cultures), responsible for 94% of culture-positive infections. Previous studies suggested *S. aureus*, followed by *S. pyogenes*, was the leading cause of cervical lymphadenitis [10, 18, 21–23, 25, 29, 30]. However, in our study, *S. aureus* comprised more than the 37.5%–77.8% range of culture-positive cases that other studies reported. Worley et al. found that *S. aureus* was responsible for 93% of cases of cervical lymphadenitis in infants compared with 59% in children [25]. Hence, the higher prevalence of infants (31%) can partially explain the dominance of *S. aureus* in cultures. Furthermore, we found no cases of *S. pyogenes* in our culture results. This finding might be because most patients had used beta-lactams before admission to our center, which are effective on *S. pyogenes* and cause negative culture results. Moreover, most of the other oral antibiotics that patients had used before hospitalization were effective on other potential etiologies of cervical lymphadenitis. Considering the relatively low prevalence of these etiologies, antibiotic use might partially explain their absence in cultures.

When choosing the empirical antibiotic in lymphadenitis, covering MRSA is vital. Studies looking at the data from 1999 to 2010 found that the prevalence of MRSA had increased in pediatric neck infections [19, 31, 32]. As a result, clindamycin was considered the first-line treatment for pediatric cervical infections [32]. With the widespread use of clindamycin, its resistance increased: Walker et al. considered clindamycin resistance an established pattern in their study [19]. Further, a study on *S. aureus* susceptibility patterns in pediatric populations found that clindamycin resistance in methicillin-susceptible *S. aureus* (MSSA) has increased and suggested pediatricians use clindamycin cautiously as empiric treatment of *S. aureus* infections [33]. In 2017, Fellner et al. found a high prevalence of clindamycin resistance despite MRSA prevalence remaining stable [21].

Clindamycin is widely used in treating cervical lymphadenitis because it covers Streptococci, especially *S. pyogenes*, which presumably causes cervical lymphadenitis. However, as we saw in this study, *S. pyogenes* was not isolated in cultures. Moreover, clindamycin, with 12% resistance, was used as the inpatient treatment in 90% of the cases, whereas cotrimoxazole and oxacillin, with 6% resistance, were used in only 8.8% of the patients. Therefore, cotrimoxazole or oxacillin may be the better empirical treatment for cervical lymphadenitis. Similar to our study, Lindquist et al. also found a high resistance to clindamycin in cervical lymphadenitis and recommended pediatricians to choose empirical therapy considering local antibiograms and a combination of cotrimoxazole and beta-lactams [34]. Moreover, clindamycin resistance should be considered in patients not responding to antibiotics, and changing the treatment to cotrimoxazole or oxacillin is recommended before needle aspiration.

Before admission to our center, beta-lactams constituted 73% of the prescribed antibiotics. This proportion was 10% and 1% for clindamycin and cotrimoxazole, respectively. Neff et al. found that the most commonly prescribed antibiotics were in the beta-lactam class [9]. Additionally, other studies found that clindamycin was most common after beta-lactams [25, 34], which corresponds to our data.

Our study has limitations, including no access to blood test results, a small number of needle aspiration or surgical drainage cultures in our study population, low prevalence of other germs responsible for cervical lymphadenitis, possible incomplete charting of patient information, and possible higher rates of complications and resistant strains because of referral from other hospitals.

Overall, *Staphylococcus aureus* predominated over other germs in our study. We found a high clindamycin resistance among *Staphylococcus* species and a low resistance to oxacillin and cotrimoxazole.

## 5. Conclusion

Considering this pattern, we recommend oxacillin as the first choice of empiric treatment for cervical lymphadenitis for both covering Streptococci and preventing further drug resistance. This might also lead to lower rates of complications and less need for surgery. Further studies are needed on the microbiology and resistance patterns of organisms causing cervical lymphadenitis in other areas of Iran.

## Figures and Tables

**Table 1:** Patient characteristics (*N* = 113).

	**n**		**%**

Gender			
Female	43		38
Male	70		62
Age range			
< 1 year old	35		31
1–3 years old	33		29
3–6 years old	26		23
> 6 years old	19		17
Hospitalization season			
Spring	22		19
Summer	28		25
Fall	29		26
Winter	34		30
Accompanying illnesses			
Upper respiratory tract infection	38		34
Dental caries	2		1.7
Hodgkin's lymphoma	1		0.9
None	72		63.7

	**Mean**	**St. dev.**	**Range**

Age at presentation (years)	3.5	2.9	0.25–13

**Table 2:** Lymph node needle aspiration or surgical drainage culture results (*N* = 113).

**Culture type**	**n** ** (%)**
Aerobic	17/28 (61)
*Staphylococcus aureus*	16/28 (57)
*Staphylococcus epidermidis*	1/28 (4)
Acid fast bacillus	0/17 (0)
Fungal	0/17 (0)

**Table 3:** Antibiotic resistance (*N* = 17).

**Antibiotic**	**n** ** (%)**
Penicillin	16 (94)
Erythromycin	2 (12)
Clindamycin	2 (12)
Oxacillin	1 (6)
Cotrimoxazole	1 (6)
Vancomycin	1 (6)

## Data Availability

The datasets used and/or analyzed during the current study are available from the corresponding author upon reasonable request.
